# Complete Genome Sequence of *Rehmannia Mosaic Virus* Infecting Rehmannia glutinosa in Japan

**DOI:** 10.1128/MRA.01115-20

**Published:** 2021-01-07

**Authors:** Naoki Komiyama, Kohei Sakuta, Masayuki Mikage, Hirosuke Shinohara, Toru Iwanami, Hiromitsu Negishi, Ok-Kyung Kim

**Affiliations:** a Department of Agriculture, Faculty of Agriculture, Tokyo University of Agriculture, Atsugi, Kanagawa, Japan; b Department of Bioresource Development, Faculty of Agriculture, Tokyo University of Agriculture, Atsugi, Kanagawa, Japan; KU Leuven

## Abstract

The complete genome sequence of isolate Jiou of rehmannia mosaic virus (ReMV) infecting Rehmannia glutinosa in Japan was obtained via Sanger sequencing. Isolate Jiou shared high nucleotide sequence identity (>94%) with other known ReMV isolates.

## ANNOUNCEMENT

Rehmannia glutinosa (Gaertn.) Libosch. ex Fisch. et C. A. Mey. is an important herbaceous medicinal plant cultivated in Asia, including China, South Korea, and Japan. The plant is commonly propagated by root division, which may facilitate the spread of viral infections. Tobacco mosaic virus (TMV), of the genus *Tobamovirus* in the family *Virgaviridae*, having a single-stranded, positive-sense RNA genome of about 6.4 kb, is one of the most destructive viruses, affecting the production of *R*. *glutinosa* quantitatively as well as qualitatively (1). Furthermore, coinfections with rehmannia mosaic virus (ReMV) and youcai mosaic virus (YoMV), both also belonging to the genus *Tobamovirus*, have been reported from *R*. *glutinosa* plants with and without symptoms in South Korea (2).

In November 2017, symptomless leaves of a single *R. glutinosa* plant were collected in Ishikawa Prefecture, Japan. Coinfections of ReMV, TMV, and YoMV were revealed by cloning and sequencing of amplicons in addition to reverse transcription-PCR (RT-PCR) with a commercial PCR primer for tobamovirus (Agdia, Inc., Elkhart, IN, USA). Here, we focused on determining the complete ReMV genome sequence because this is the first report of ReMV infecting *R*. *glutinosa* naturally in Japan.

After three successive single-lesion isolations from Chenopodium amaranticolor, the virus isolate was named Jiou and further propagated in Nicotiana benthamiana (3, 4). Total RNA was extracted from 0.1 g of the infected N. benthamiana leaves using TRIzol reagent (Thermo Fisher Scientific, Inc., Waltham, MA, USA) and used as input for RT-PCR. The RT-PCR was performed with 5 pairs of primers newly designed in this study (Table 1), using the ReverTra Ace (Toyobo Co., Ltd., Osaka, Japan) and *Ex Taq* (TaKaRa Bio, Inc., Shiga, Japan) kits. The nucleotide sequences from both ends of the genome were determined by rapid amplification of cDNA ends (RACE) using a SMARTer RACE 5′/3′ kit (TaKaRa Bio, Inc.) and the 3′ RACE system (Invitrogen, Carlsbad, CA, USA). The PCR products were purified using the Wizard SV gel and PCR clean-up system (Promega Corp., Madison, WI, USA), ligated into a pGEM-T-vector (Promega Corp.), and then transformed into Escherichia coli strain JM109 (TaKaRa Bio, Inc.). Plasmid DNA was isolated from at least 3 independent clones using a Labo Pass minikit (Cosmo Genetech Co., Ltd., Seoul, South Korea) and sequenced in an ABI 3730xl DNA analyzer (Applied Biosystems, Foster City, CA, USA) by Macrogen Japan Corp. (Kyoto, Japan). The complete nucleotide sequence was assembled from the consensus sequence of at least 3 clones for the target region using BioEdit software (5).

**TABLE 1:** List of primers used to amplify the rehmannia mosaic virus genome sequence in this study

Primer^*a*^	Sequence (5′ to 3′)	Amplification region(s)^*b*^	Amplicon length (bp)
ReMV-5RACE440	CGTATGCCCGTCCTTTGAACAGATGCG	5′ UTR and partial ORF1	438
ReMV-5RACE	YTTRCAAAACCARGTRTTAACCCTGG	5′ UTR and partial ORF1	998
ReMV-jiou5F	TTTCCACTTCGCCGAAGGTC	Partial ORF1	1,517
ReMV-jiou5R	TAGCAGCAGTATCCTTTAGG		
ReMV-3F	CGAGGAAATTGARTCATTGG	Partial ORF1	1,212
ReMV-3R	AGAGCAACCAAAACATGAGG		
ReMV-1F	TGAGGTGCAAGGAGAGACG	Partial ORF1 and partial ORF2	1,104
ReMV-1R	CTCCGCCAGATTTCGTACTC		
ReMV-2F	CGAAAGGRTGYGAGTTCCC	Partial ORF2 and partial MP	996
ReMV-2R	TCTGACAGACATTGGAACGTC		
ReMV-6F	ACCCATGGAACTCACCGA	Partial MP and CP and partial 3′ UTR	796
ReMV-6R	GTTATCGTACGCACCACG		
ReMV-3RACE400	AGGCGAACCCTACAACTGCTGAAACG	Partial CP and 3′ UTR	386

a The capital letters F and R at the end of primer names indicate, respectively, the forward and reverse primers used in the RT-PCR amplification described in the text.

b UTR, untranslated region; MP, movement protein; CP, coat protein.

**FIG 1:**
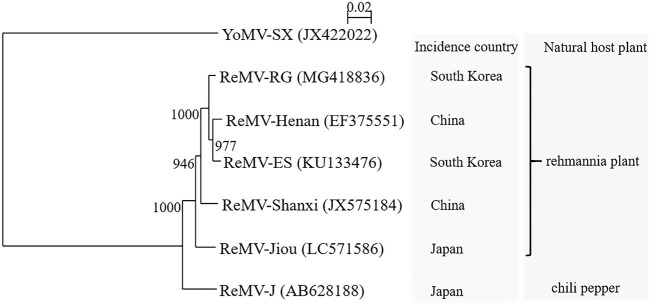
Phylogenetic tree obtained using the neighbor-joining (NJ) method based on the complete nucleotide sequences of the rehmannia mosaic virus (ReMV) isolates with youcai mosaic virus (YoMV) as the outgroup. Their accession numbers are indicated in parentheses. Multiple sequence alignments were generated by using ClustalW version 2.1 with default parameters (7) on the DNA Data Bank of Japan (DDBJ) Web server. The tree was visualized using the program NJplot. The numbers at the nodes represent the bootstrap support values for 1,000 replicates in the tree. The scale bar above the tree represents the number of nucleotide substitutions per site. The incidence countries and natural host plants are shown on the right.

The complete genome sequence of isolate Jiou (GenBank accession number LC571586) was 6,395 nucleotides (nt) long (GC content, 43.22%). A direct BLASTn search in the NCBI nucleotide database showed the highest nucleotide sequence identity (97%) with the ReMV isolate Shanxi (JX575184). Four open reading frames (ORFs) were predicted by the NCBI ORFfinder (https://www.ncbi.nlm.nih.gov/orffinder/). ORF1, which encoded a 126-kDa replicase protein, spanned from nucleotides 72 to 74 (AUG start codon) to nucleotides 3420 to 3422 (amber UAG stop codon) and translated into 1,116 amino acids (aa). As in other tobamoviruses, the CAA and UUA codons (nucleotides 3423 to 3428) were found downstream of the UAG stop codon, which may promote a read-through of the leaky terminator in ORF1 (6). Consequently, ORF2 (nucleotides 72 to 4922), which encoded a 183-kDa RNA-dependent RNA polymerase (RdRp), was predicted. ORF3 (nucleotides 4906 to 5709) encoded a 30-kDa movement protein, and ORF4 (nucleotides 5712 to 6191) encoded an 18-kDa coat protein. The 5′ and 3′ untranslated regions contained 71 and 204 nt, respectively. Isolate Jiou shared 96 to 97% identity with 2 Korean isolates (KU133476 and MG418836) and 2 Chinese isolates (EF375551 and JX575184) from rehmannia plants and 94% identity with 1 Japanese chili pepper isolate (AB628188). Pairwise comparison and phylogenetic analysis showed a closer relationship between isolate Jiou and isolates from the same host plant than between isolates collected from the same country (Fig. 1).

Necrotic and mosaic symptom-inducing ReMV from chili pepper plants has been reported in Japan (4), but this is the first report of ReMV naturally infecting *R. glutinosa*.

### Data availability.

The complete genome sequence of the ReMV isolate Jiou has been deposited in GenBank under accession number LC571586.
